# Comprehensive promotion of drug traceability codes in China in 2025: challenges and solutions for tertiary outpatient pharmacists

**DOI:** 10.3389/fphar.2025.1619916

**Published:** 2025-07-25

**Authors:** Zhuo Zhao, Haozheng Wang, Junyi Zhai, Zheng Wang

**Affiliations:** ^1^Key Laboratory of Shaanxi Province for Craniofacial Precision Medicine Research, College of Stomatology, Xi’an Jiaotong University, Xi’an, China; ^2^Clinical Research Center of Shaanxi Province for Dental and Maxillofacial Diseases, College of Stomatology, Xi’an Jiaotong University, Xi’an, China; ^3^State Key Laboratory for Manufacturing System Engineering, Xi’an Jiaotong University, Xi’an, China; ^4^Medical Insurance Administration Office, College of Stomatology, Xi’an Jiaotong University, Xi’an, China; ^5^Medical Quality and Safety Management Office, College of Stomatology, Xi’an Jiaotong University, Xi’an, China; ^6^Department of Pharmacy, College of Stomatology, Xi’an Jiaotong University, Xi’an, China

**Keywords:** drug traceability codes in China healthcare policy implementation, drug traceability code, outpatient pharmacist, real-world experience, China

## Abstract

Medicines are a highly regulated category of commodities that demand strict oversight across their distribution chains and face numerous challenges in ensuring safety and authenticity. In response to concerns about counterfeit products, gray-market diversion, and pressures to protect public insurance funds, China has rapidly advanced a nationwide drug traceability policy, mandating unit-level digital identifiers and requiring point-of-dispensing scanning by 1 July 2025. However, given China’s vast territory, substantial regional disparities, and complex healthcare infrastructure, this accelerated policy rollout has posed considerable challenges for frontline implementation, placing significant pressure on outpatient pharmacy operations. In high-volume tertiary hospitals, where prescription volumes are exceptionally large, the manual scanning of traceability codes has markedly increased dispensing times, prolonged patient waiting, and heightened pharmacist workloads, further complicated by inconsistent barcode placement and hardware limitations. To explore potential solutions, our team developed and evaluated a machine vision–based scanning prototype under laboratory conditions. The system demonstrated meaningful workflow improvements in simulated dispensing scenarios and holds promise for future validation and adaptation in real-world settings. Drawing on our experiences as frontline pharmacists, this study provides practical observations from a tertiary hospital in Northwestern China, examining dispensing processes before and after traceability code integration. We hope these findings contribute to international dialogue on pharmaceutical management, helping to advance medication safety, governance, and equitable access in healthcare systems worldwide.

## Graphical Abstract

**Figure:**
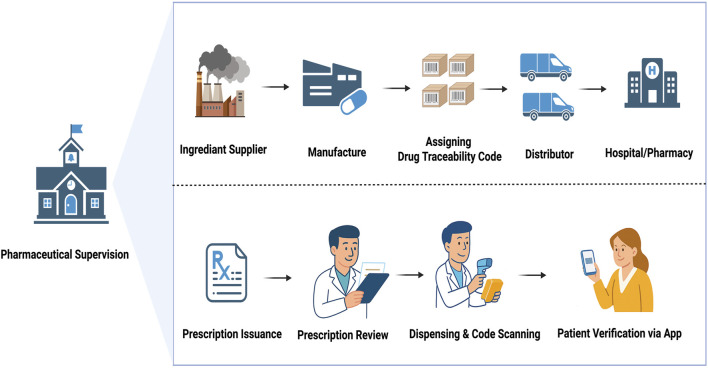
To combat counterfeit and diverted drugs, the Chinese government will mandate traceability code scanning for all reimbursed prescriptions beginning 1 July 2025. The visual illustrates the supervised traceability workflow: from manufacturing with code assignment, through distribution to hospitals and pharmacies, where pharmacists conduct prescription review, dispensing, and code scanning. Patients can verify authenticity via mobile applications. While this “one drug, one code” system enhances pharmaceutical safety and transparency, its implementation faces challenges in China’s large and unevenly developed regions.

## 1 Introduction

Ensuring that genuine and safe medications reliably reach patients remains a major challenge across the pharmaceutical pipeline, from initial discovery through to regulatory authorization (Kannarkat et al., 2024; Omidian et al., 2023; Tobias et al., 2021; Hamilton et al., 2016; Klein and Stolk, 2018). In recent years, China’s healthcare system has faced escalating challenges in pharmaceutical regulation, particularly surrounding drug diversion, resale, and the circulation of counterfeit medications (Simborg, 2014; Mackey et al., 2020). These issues expose deeper vulnerabilities in drug supply chain governance, especially in a vast and economically diverse country where regional disparities in medical insurance reimbursement policies create opportunities for arbitrage. For instance, acarbose tablets (Glucobay^®^) may retail for as little as CNY 6 (USD 0.85) in Beijing due to generous local reimbursement, but are often resold for CNY 30–50 (USD 4.20–7.00) in underdeveloped regions where coverage is limited or absent. Such loopholes have fostered a thriving gray market, jeopardized drug authenticity and patient safety, and undermined the financial sustainability of national insurance funds (Lawson and Rodriguez, 2016; Kelesidis and Falagas, 2015; Mackey et al., 2015).

To address these risks, China’s National Healthcare Security Administration (NHSA) launched a nationwide drug traceability initiative in 2024. The policy mandates the use of unique traceability codes on each drug package, which must be scanned in real-time at the point of sale to validate medical insurance claims (Ho et al., 2020; Richardson et al., 2012; Bach et al., 2015). Beginning 1 July 2025, prescriptions that fail to capture traceability codes will not be eligible for reimbursement. By 1 January 2026, full traceability data collection will become mandatory for all designated healthcare providers (Jessica et al., 2024; Hokanson et al., 2025). While this initiative marks a significant step toward digitizing and modernizing pharmaceutical oversight (Wen et al., 2025; Uddin et al., 2021; Gomasta et al., 2023; Haji et al., 2021; Munasinghe and Halgamuge, 2023; Mani et al., 2022), its rapid rollout has presented new operational burdens for frontline personnel—particularly in high-volume public hospitals.

These challenges are especially pronounced in tertiary hospitals located in provincial capitals, where centralized healthcare infrastructure concentrates patient demand (Hamilton et al., 2016; Klein and Stolk, 2018; Portela et al., 2017). A typical outpatient pharmacy in such settings operates over ten dispensing windows, each handling 800–1,000 prescriptions per day (Gao et al., 2024; Bu et al., 2022; Gil-Candel et al., 2023; Chapuis et al., 2010). The implementation of the traceability policy requires pharmacists to manually scan each drug package using handheld barcode scanners. Outpatient prescriptions often include multiple medications, and the traceability codes are inconsistently positioned on the packaging, necessitating repeated rotation and adjustment of each item to locate the correct code. This process significantly increases the time and labor involved in dispensing, lengthening patient wait times and intensifying pharmacist workload. These operational frictions highlight the need for more efficient technical tools that can support policy enforcement without compromising frontline service capacity.

As practicing outpatient pharmacists in a densely populated, resource-limited environment, we have witnessed firsthand how the rapid rollout of traceability requirements has transformed daily pharmacy operations in China (Graphical Abstract). Drawing on our frontline experience and simulation-based workflow testing conducted in a university-affiliated laboratory, we introduce a prototype machine vision–based scanning system designed to accelerate code recognition and reduce manual handling. Through this grounded account, here we aim to share practical insights into how digital innovations are being explored and adapted in countries like China to modernize pharmaceutical governance—balancing regulatory ambition with real-world feasibility in the era of intelligent healthcare.

## 2 The drug traceability codes system in China: structure, policy design, and enforcement mechanisms

**FIGURE 1:**
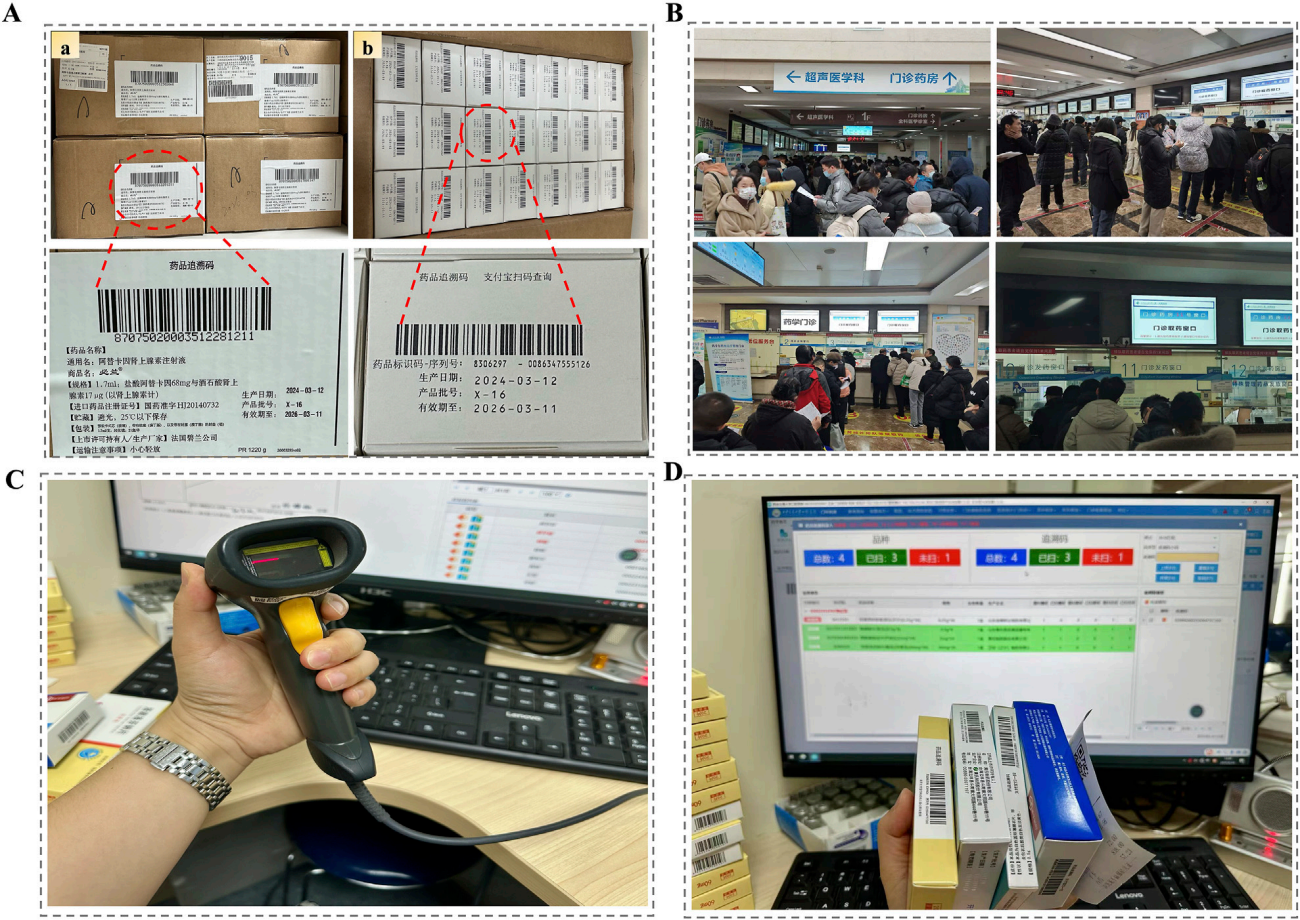
Real-World Implementation of Drug Traceability Codes in a High-Volume Tertiary Hospital Pharmacy in China. **(A)** Examples of outer and retail-level traceability codes on pharmaceutical packages; **(B)** Crowded outpatient pharmacy counters with high daily prescription volumes; **(C)** The handheld barcode scanner currently used for manual scanning of traceability codes; **(D)** Pharmacy information system interface displaying code-captured medication records during dispensing.

China’s drug traceability system is a nationwide digital infrastructure designed to ensure full-process supervision over the production, distribution, and use of pharmaceuticals (Omidian et al., 2023). Built on the principle of “one product, one code,” the system applies a three-tier coding structure: large codes for batch tracking at the manufacturer and distributor level, medium codes for hospital or pharmacy warehouse intake, and small codes—affixed to individual retail units—for final dispensing and insurance reimbursement (Figure 1A). Since 2024, all nodes in the supply chain are required to scan the appropriate level of code upon entry and exit (Poon et al., 2010; Pereira et al., 2012; Ramakanth et al., 2021; Jarrett et al., 2020; Sagi et al., 2017). In clinical settings, pharmacists scan small codes in real time at the point of dispensing, while logging into personal accounts registered with the National Health Commission. Patients likewise access medication via their unique medical insurance IDs. This dual authentication establishes a fully traceable and accountable drug flow between producer, provider, and patient (D’Anna et al., 2022; Griffin et al., 2019).

**FIGURE 2:**
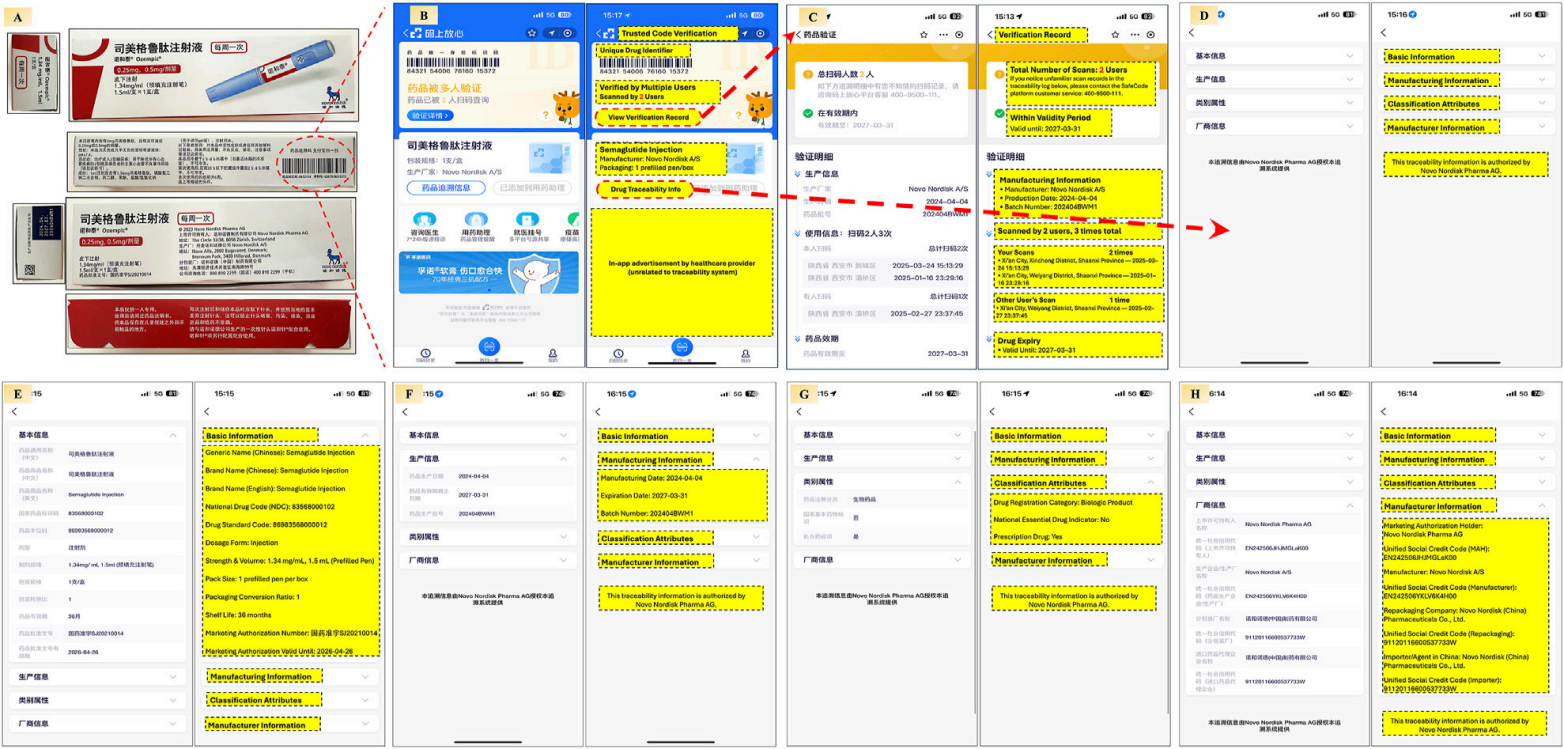
Patient-oriented end-to-end drug traceability verification using China’s digital traceability system via the Alipay app This figure demonstrates how patients can independently verify medication authenticity and traceability through China’s national drug traceability platform embedded in Alipay. **(A)** Semaglutide injection packaging showing the tertiary code on the carton and a primary code on each retail unit; **(B)** The Alipay app’s main verification screen, displaying trusted code validation, scan history, manufacturer information, packaging specifications, and traceability details; **(C)** Verification Record including number of scans, expiration validation, and scan history; **(D)** Section directory linking to Basic Information, Manufacturing Information, Classification Attributes, and Manufacturer Information; **(E)** Basic Information including generic/brand names, national drug codes, packaging specifications, dosage, and shelf life; **(F)** Manufacturing Information covering production date, batch number, and expiry; **(G)** Classification Attributes including regulatory category, essential drug indicator, and prescription status; **(H)** Manufacturer Information details with marketing authorization holder, manufacturer, re-packager, and local agent, including their registration codes.

In real-world practice, Alipay, one of China’s most widely used mobile platforms, offers an integrated drug traceability feature. By scanning the code via the app, users can instantly access information such as registration details, distribution records, and regulatory status. This empowers patients to verify authenticity, builds consumer confidence, and fosters a transparent and trustworthy pharmaceutical environment (Figure 2). Scanning the small code enables users to track the origin and distribution path of each unit, thereby achieving full-chain traceability.

Unlike many high-income countries (Lober et al., 1988; Shahbahrami et al., 2024; Tseng et al., 2018; Klein et al., 2020; Klein et al., 2016; Akram et al., 2024; Headquarters United States Air Force Surgeon General, 2024), where traceability codes are used primarily for recalls or supply chain audits, China’s system is embedded directly into daily pharmacy operations and medical insurance settlement (Shen et al., 2024; Edwards et al., 2020; Sood et al., 2021; Wang and Jie, 2020; Chukwu et al., 2017; Socal et al., 2021). For instance, in the United States, the Drug Supply Chain Security Act (DSCSA) requires serialization and transactional data sharing, but does not mandate code scanning during dispensing. In contrast, China links traceability directly to insurance reimbursement: starting July 2025, any prescription lacking successful traceability code capture will be denied reimbursement, and by January 2026, full traceability data submission will be mandatory nationwide. This level of integration—combining pharmaceutical safety with financial oversight and real-time individual-level tracking—is both ambitious and unprecedented, especially in a country with vast regional disparities in healthcare infrastructure.

To further strengthen oversight, recent policy updates have extended coding requirements beyond commercial drugs (Coleman, 2012). Hospital-prepared formulations and traditional Chinese patent medicines are now subject to traceability mandates. The only current exemption applies to injectable drugs dispensed in split doses within hospitals. Together, these measures reflect a regulatory vision that aims not only to eliminate counterfeit and diverted drugs, but also to lay a digital foundation for precision reimbursement, supply chain transparency, and intelligent health governance. However, this vision also introduces new demands on pharmacy workflows, which will be examined in the next section (Yulia et al., 2025; Slowiak and Huitema, 2015; Sun et al., 2017).

## 3 Implementation barriers in a stratified healthcare system: from tertiary hospitals to rural clinics

The rollout of China’s drug traceability code policy has exposed significant disparities across different tiers of the healthcare system. While the initiative aspires to establish end-to-end pharmaceutical accountability, its practical implementation—particularly in high-volume tertiary hospitals and resource-limited grassroots clinics—has proven far more complex than initially anticipated (Richardson et al., 2012; Yulia et al., 2025).

In large public hospitals, especially those in provincial capitals, outpatient pharmacies face tremendous daily workloads (Slowiak and Huitema, 2015; Sun et al., 2017). A typical tertiary hospital may operate more than ten dispensing windows, each processing between 800 and 1,200 prescriptions per day, with cumulative volumes often exceeding 10,000 (Hammoudeh et al., 2021). As shown in Figure 1B, long lines of patients waiting at pharmacy counters are commonplace. Under China’s institutional workflow, physicians issue prescriptions, pharmacists conduct mandatory prescription auditing, and only then proceed to medication dispensing. The traceability policy introduces an additional verification step at the dispensing stage, where pharmacists must scan the code on each package before release (Figures 1C,D). This seemingly minor change has introduced notable operational frictions. Traceability codes are printed on varying parts of the package—side panels, bottoms, or curved flaps—making them difficult to locate quickly. As outpatient prescriptions frequently include multiple medications (Alam et al., 2018; Ciapp et al., 2021; Steinman, 2019; Foot et al., 2022), pharmacists must rotate and inspect each box to find the code, significantly increasing the time required per patient. Standard barcode scanners can process only one item at a time and require precise alignment, adding further inefficiency. These cumulative delays have led to longer queues, extended wait times, and mounting patient dissatisfaction—particularly during peak hours (Yulia et al., 2025; Alodan et al., 2020).

Compounding this pressure is China’s “zero mark-up” drug pricing policy, which prohibits hospitals from earning profit on medication sales. This means hospitals cannot recoup the cost of added labor by expanding their pharmacy workforce. As a result, the increased burden of traceability code scanning falls entirely on existing staff. Over time, this intensifies physical fatigue from repetitive handling and mental strain from sustained concentration, raising the risk of dispensing errors and impacting pharmacist wellbeing (Chapuis et al., 2010; Ciapp et al., 2021).

In contrast, grassroots healthcare facilities—such as township health centers and rural clinics—face challenges of a different nature. These institutions often lack the digital infrastructure required to support traceability compliance. Barcode scanners may be obsolete or missing, staff may have limited technical training, and internet connectivity is often unstable. In such settings, manual entry is prone to delays and errors, and real-time data submission is often not feasible. These barriers are not merely logistical—they reflect deeper structural inequalities in China’s healthcare delivery system. The so-called “last-mile” gap between policy goals and local implementation is especially stark in these under-resourced regions.

Taken together, these challenges highlight the need for differentiated, tier-specific solutions. Tertiary hospitals require automation tools that can streamline high-throughput pharmacy operations, while grassroots clinics need affordable, easy-to-use systems supported by infrastructure investment and capacity-building efforts. Without such tailored implementation strategies, the national traceability policy may fall short of its intended impact—and could unintentionally widen existing healthcare disparities.

## 4 Machine vision-based integrated systems: an innovative solution

**FIGURE 3:**
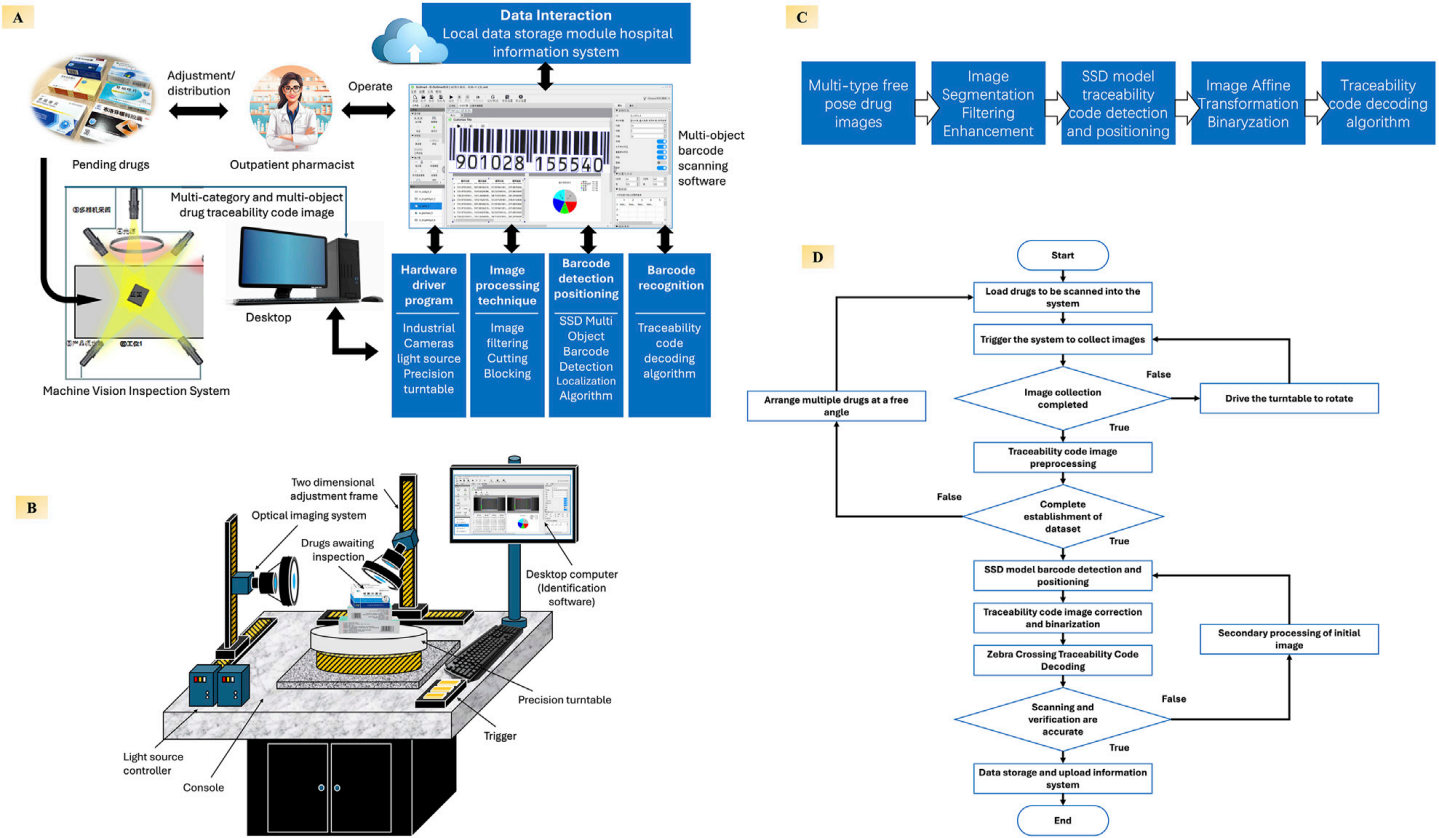
Architecture, workflow, and core algorithms of the proposed machine vision–based drug traceability code scanning system **(A)** System overview showing how outpatient pharmacists load medication packages into the inspection platform, where multi-angle images of traceability codes are automatically captured. Recognized traceability data are stored locally and synchronized with the hospital information system to enable full-chain traceability management; **(B)** Hardware layout depicting the scanning station, including industrial cameras, dynamic light sources, precision turntable, and user-facing workstation interface; **(C)** Core image processing pipeline, including image segmentation, enhancement, SSD-based barcode positioning, affine transformation, binarization, and decoding algorithms for reliable recognition; **(D)** Operating flowchart of the system, describing the entire sequence from drug loading and image acquisition to data preprocessing, barcode detection, and data upload.

In recent years, machine vision technology has been widely adopted across diverse industries such as manufacturing, logistics, and quality inspection, enabling high-throughput, precise, and automated recognition capabilities (Bluethgen et al., 2024). Within healthcare, machine vision has already shown promising results in applications including virtual staining of tissue samples and digital pathology slide interpretation, enhancing efficiency and reducing human error (Peng et al., 2025). Inspired by these advances, we explored its potential in pharmaceutical management by independently developing a machine vision–based system specifically designed for drug traceability code recognition which enables high-throughput, accurate, and ergonomically efficient barcode scanning in pharmacy settings (Zheng et al., 2023). This prototype was designed and evaluated in a university-affiliated laboratory, aiming to support both tertiary hospitals and grassroots healthcare institutions through scalable, intelligent automation (Figure 3A).

At its core, the system integrates a multi-angle barcode recognition module, incorporating high-resolution industrial cameras, adaptive lighting controls, and parallelized image-processing algorithms to automatically detect and decode traceability codes from multiple surfaces of pharmaceutical packaging (Figure 3B). Unlike conventional handheld scanners that require pharmacists to manually rotate and align each box, the system supports batch scanning of randomly oriented packages without extra manipulation, substantially reducing both labor intensity and processing time. The platform can scan up to 20 drug packages in a single capture cycle while delivering structured traceability data in real time. To address the needs of infrastructure-limited or remote settings, the system incorporates offline functionality and local data caching. In environments with poor or unstable network connections—such as township health centers or rural clinics—traceability data can be stored locally and uploaded once connectivity is restored, ensuring continued compliance with national traceability requirements without interrupting dispensing workflows. The overall image-processing pipeline developed by our team is summarized in Figure 3C. As shown in Figure 3D, once the packages are loaded and the system is triggered, it automatically proceeds through image acquisition, preprocessing, barcode detection using an SSD model, code decoding with Zebra Crossing algorithms, and data submission, requiring minimal operator input and thus relieving frontline pharmacists of repetitive scanning tasks.

In a standardized evaluation (Table 1), dispensing times without code scanning and with handheld barcode scanning were measured in routine outpatient pharmacy practice, while data for the machine vision–based platform were obtained from laboratory-based testing designed to replicate realistic medication layouts. Under these conditions, the current handheld barcode scanning workflow increased average dispensing time to approximately 154.4% of the baseline (no-scanning) scenario, reflecting significant delays (*P* < 0.05). In contrast, the machine vision–based system reduced this burden to 116.5% of baseline (*P* < 0.05), indicating a substantial improvement in operational efficiency. This machine vision platform is currently under substantive examination for patent approval in China.

**TABLE 1:** Comparison of dispensing times for representative outpatient medication scenarios under three conditions: without code scanning, with handheld barcode scanning, and a machine vision–based platform.

Representative outpatient medication scenarios		Dispensing time (seconds)		
Diagnosis	Medication Details	Without Code Scanning	Handheld Barcode Scanner	Machine Vision–Based Platform
Recurrent Aphthous Ulcers	• Bovine Basic Fibroblast Growth Factor Gel ×1 • Thalidomide Tablets ×1 • Compound Chamomile and Lidocaine Gel ×1 • Cetylpyridinium Chloride Lozenges ×1	45	69	48
Chronic Periodontitis	• Minocycline Hydrochloride Ointment ×1 • Cetylpyridinium Chloride Lozenges ×1 • Articaine with Epinephrine Injection ×1 • Lidocaine Hydrochloride Injection ×1 • Compound Chlorhexidine Mouthwash	42	65	51
Impacted Tooth	• Amoxicillin Capsules ×1 • Metronidazole Tablets ×1 • Loxoprofen Sodium Tablets ×1 • Compound Chlorhexidine Mouthwash ×1	48	70	55
Chronic Periapical Periodontitis	• Tinidazole Tablets ×1 • Compound Chlorhexidine Mouthwash ×1 • Methylene Blue Injection ×1	23	40	30
Overall average		100%	154.4%^a^	116.5%^#^

Notes: All prescriptions were drawn from common oral and dental conditions treated at the largest stomatological hospital in Northwest China. Dispensing times were measured 20 times per prescription under three conditions: no scanning, handheld barcode scanning, and a laboratory-simulated machine vision–based batch scanning platform. Counseling steps were excluded from timing measurements. The bottom row shows average dispensing times expressed as proportions of the no-scanning baseline (set to 100%): handheld scanning significantly increased dispensing time by 54.4% (*P* < 0.05), while the machine vision platform reduced this burden to 116.5% of baseline (*P* < 0.05). These data support a patent application currently under substantive examination.

^a^ Dispensing time using handheld barcode scanner vs. Dispensing time without code scanning; # Dispensing time using machine vision–based platform vs. Dispensing time without code scanning.

From an engineering and economic perspective, the prototype’s total development cost was approximately RMB 100,000 (about USD 13,700), including hardware, software, and system integration. With mass production, the unit price could potentially be reduced below RMB 50,000 (about USD 6,800), which, although higher than conventional handheld scanners, would likely be acceptable for large tertiary hospitals with extremely high prescription volumes and limited staffing flexibility. For rural or township clinics, the system’s offline capability could help bridge connectivity gaps, though its overall cost-effectiveness in these settings still requires further evaluation. The platform is currently under substantive patent examination in China and has not yet been commercialized, with additional real-world validation needed before broader adoption. Applying machine vision to address the practical challenges of traceability code promotion in China appears to hold considerable promise, offering an intelligent, scalable, and policy-aligned solution that may help strengthen pharmaceutical governance. Nevertheless, the current findings are based on laboratory simulations and limited real-world workflow observations, which may not fully capture the complexity of China’s vast, densely populated, and regionally diverse healthcare system. Although laboratory testing has been encouraging, the system has yet to undergo clinical validation, and its real-world performance, acceptability, and sustainability remain to be confirmed. Broader pilot studies in authentic medical settings will be crucial for further optimization and validation.

## 5 Conclusion and future directions

As pharmacists directly impacted by these policy changes, we have provided an overview of China’s drug traceability code framework, highlighted the practical challenges faced in daily pharmacy operations, and presented our team’s development of a machine vision–based scanning system as a potential solution. While initial laboratory tests have been encouraging, future work must include thorough validation in real-world clinical environments to optimize performance and assess long-term feasibility. Looking ahead, we believe China’s pharmaceutical governance will continue to improve through such innovative efforts, and we are committed to refining and implementing our system in practical settings to help ensure safer, more transparent, and more equitable medication use in the future.
